# Supplementary material for: The Systems Biology Research Tool: evolvable open-source software
Source: BMC Syst Biol. 2008 Jun 29;2:55. doi: 10.1186/1752-0509-2-55 (PMC2446383; doi:10.1186/1752-0509-2-55)
Supplement: Additional file 1 — SBRT Archive. An archive of the current version of the Systems Biology Research Tool. [file 1752-0509-2-55-S1.zip › sbrt-1.4.0/doc/developers_guide/api/sbrt/kernel/io/AbstractResultsManager.html]

AbstractResultsManager


|  |  |  |
| --- | --- | --- |
| **Overview** \| **Package** \| **Class** \| **Use** \| **Tree** \| **Deprecated** \| **Index** \| **Help** | |  |
| PREV CLASS   **NEXT CLASS** | **FRAMES**    **NO FRAMES**     **All Classes** |  |
| SUMMARY: NESTED \| FIELD \| CONSTR \| METHOD | DETAIL: FIELD \| CONSTR \| METHOD |  |


---


## sbrt.kernel.io Class AbstractResultsManager<R>

```
java.lang.Object
  sbrt.kernel.io.AbstractResultsManager<R>
```

**Type Parameters:**: `R` - the result type.

**All Implemented Interfaces:**: ResultsManager<R>

**Direct Known Subclasses:**: ExtremeCurrentManager.Rm, ListResultsManager, SingleRm

---

``` public abstract class AbstractResultsManager<R> extends java.lang.Object implements ResultsManager<R> ```

This class is a skeleton implemenation of `ResultsManager`.

**Author:**
:   This class was written and documented by Jeremiah Wright
    while in the Wagner lab.

---

**Constructor Summary**

`AbstractResultsManager()`
  
          Constructs a new results manager.


| **Method Summary** | |
| --- | --- |
| `void` | `breakPoint()`             Does nothing. |
| `protected  void` | `checkState()`             Ensures `close()` has not already been called. |
| `void` | `close()`             Closes this results manager. |
| `java.lang.String` | `getName()`             Returns an empty string. |
| `boolean` | `isClosed()`             Indicates if this results manager has already been closed. |

**Methods inherited from class java.lang.Object**

`clone, equals, finalize, getClass, hashCode, notify, notifyAll, toString, wait, wait, wait`

**Methods inherited from interface sbrt.kernel.io.ResultsManager**

`addData`

**Constructor Detail**

### AbstractResultsManager

```
public AbstractResultsManager()
```

:   Constructs a new results manager.


**Method Detail**

### breakPoint

```
public void breakPoint()
```

:   Does nothing.

    :   **Specified by:**: `breakPoint` in interface `ResultsManager<R>`

---


### close

```
public void close()
```

:   Closes this results manager. If already closed,
    invoking this method has no effect.

    :   **Specified by:**: `close` in interface `ResultsManager<R>`

---


### isClosed

```
public boolean isClosed()
```

:   Indicates if this results manager has already been closed.

    :   **Specified by:**: `isClosed` in interface `ResultsManager<R>`
    :   **Returns:**: `true` if this results manager is closed; `false` othewise. **See Also:**: `ResultsManager.close()`

---


### getName

```
public java.lang.String getName()
```

:   Returns an empty string.

    :   **Specified by:**: `getName` in interface `ResultsManager<R>`
    :   **Returns:**: an empty string.

---


### checkState

```
protected void checkState()
```

:   Ensures `close()` has not already been called.

    :   **Throws:**: `java.lang.IllegalStateException` - if `close()` has been called.


---


|  |  |  |
| --- | --- | --- |
| **Overview** \| **Package** \| **Class** \| **Use** \| **Tree** \| **Deprecated** \| **Index** \| **Help** | |  |
| PREV CLASS   **NEXT CLASS** | **FRAMES**    **NO FRAMES**     **All Classes** |  |
| SUMMARY: NESTED \| FIELD \| CONSTR \| METHOD | DETAIL: FIELD \| CONSTR \| METHOD |  |


---
